# Incidence and Risk Factors for Short-Term Reoperations After Open First-Metatarsal Osteotomy for Hallux Valgus

**DOI:** 10.1177/24730114251359646

**Published:** 2025-08-22

**Authors:** Tuuli Erjanti, Heli Keskinen, Tiia Rissanen, Keijo Mäkelä, Petteri Lankinen, Inari Laaksonen, Helka Koivu

**Affiliations:** 1 Department of Orthopaedics and Traumatology, Turku University Hospital and University of Turku, Turku, Finland; 2 Department of Pediatric Surgery and Orthopaedics, Turku University Hospital, Turku, Finland; 3 Department of Biostatistics, University of Turku and Turku University Hospital, Turku, Finland; 4 Hospital Pihlajalinna Turku, Turku, Finland

**Keywords:** hallux valgus, osteotomy, revision, complication, diabetes

## Abstract

**Background:**

Despite the satisfactory results of hallux valgus surgery in general, the incidence of complications has been considerably high. This study evaluated the incidence and risk factors of short-term reoperations after surgical correction of hallux valgus deformity with first metatarsal osteotomy.

**Methods:**

We conducted a retrospective review of 685 consecutive open first-metatarsal osteotomies performed from 2013 to 2018 in a single university-hospital region. The number, indications, and type of reoperation were collected. The association between reoperation and patient’s age, sex, BMI, comorbidities, preoperative hallux valgus angle (HVA), hospital type, osteotomy type, and surgeon’s experience was analyzed.

**Results:**

There were 79 reoperations (11.5%) at a median of 14 months (range, 1-83) postoperatively. Fifteen reoperations were merely hardware removals. Most reoperations (46%) were performed because of residual deformity. Preoperative and postoperative HVA, diabetes, and type of osteotomy were statistically significant risk factors for all-cause reoperation both in univariate (*P* < .0001, *P* = .0052, and *P* < .0001, respectively) and multivariate analysis (*P* < .0001, *P* = .0059, and *P* < .0001, respectively). Overall, 4.9% of distal, 18.7% of midshaft, and 29.3% of proximal osteotomies were reoperated.

**Conclusion:**

The incidence of short-term (≤24-month) reoperations was higher than previously reported after open surgical correction of hallux valgus deformity with first metatarsal osteotomy. Larger preoperative and postoperative HVA, diabetes, and type of osteotomy were associated with revision surgery in this retrospective cohort. Proximal osteotomies had the highest risk for reoperation.

**Level of Evidence:**

Level IV, case series.

## Graphical abstract

**Graphical Abstract:**
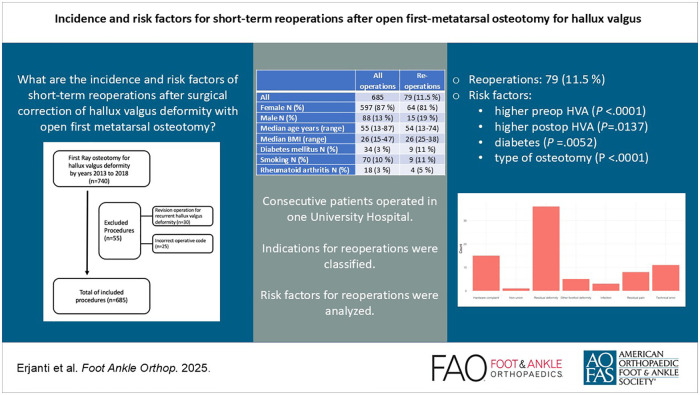

## Introduction

Hallux valgus is a common forefoot deformity affecting foot function and quality of life.^1-4^ Surgical treatment is often required, and open first metatarsal osteotomy is one of the most common primary operative procedures for hallux valgus deformity.^4-6^ Successful surgical treatment has been shown to improve the quality of life of hallux valgus patients.^7^

Despite the satisfactory results in general, the incidence of complications and recurrence has been considerably high after first metatarsal osteotomies, up to 55%.^8-11^ Further recurrence, nonunion, and reoperations are the most common complications following primary hallux valgus surgery, of which residual deformity has an incidence ranging from 3% to 16%.^10,12^ Increased preoperative hallux valgus angle (HVA) and undercorrection (postoperative HVA >15-20 degrees or incomplete sesamoid reduction) have been shown to increase the risk for the recurrence of hallux valgus.^9,13-16^ This is important clinically, as patients with radiologic undercorrection and reoperation have lower patient-reported outcome measure (PROM) scores compared with the optimal radiologic outcome and primary operations.^15^

The incidence of revision surgery after hallux valgus deformity correction for various indications to range from 0.4% up to 12.6%.^8,11,12,15,17-19^ In a previous study by Thier et al (2024), multiple comorbidities were found to correlate with higher revision rates and the odds for hallux valgus revision rates to be significantly greater in procedures performed before 2010.^11^

Although there is considerable evidence regarding the complications related to hallux valgus surgery and the risk factors for recurrence, the literature regarding the reasons and risk factors for reoperation, especially in the short term, is scarce. This study aimed to evaluate the incidence and risk factors of short-term reoperations after surgical correction of hallux valgus deformity with first metatarsal osteotomy in a large University Hospital cohort.

## Methods

### Patients

**Figure 1.**
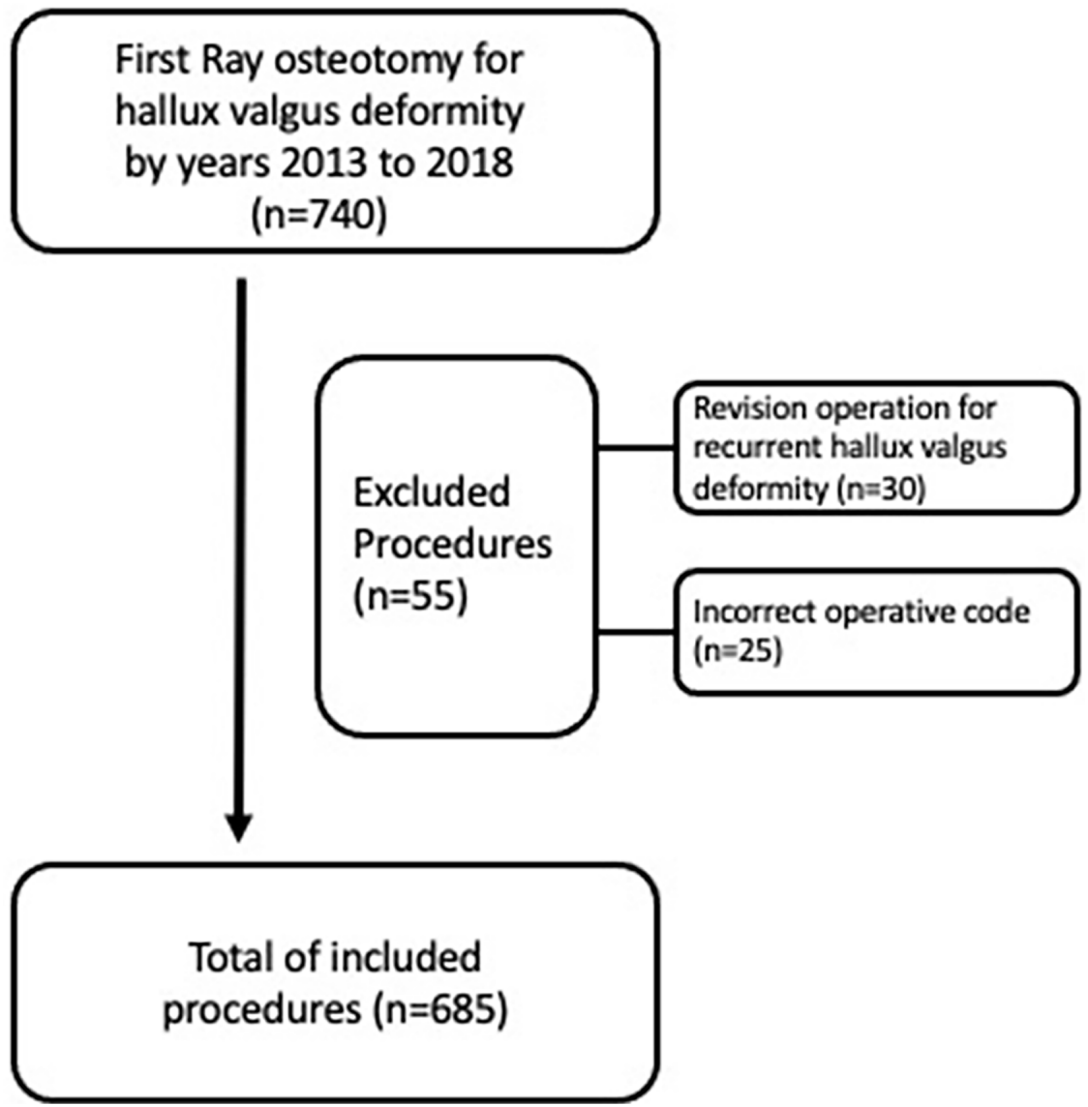
Flow chart.

Consecutive patients operated with an open first metatarsal osteotomy to correct hallux valgus deformity in one University Hospital region of half a million inhabitants, including 5 different hospitals in 2013-2018, were reviewed. The data were searched from the health care patient database with a diagnosis code for hallux valgus (*International Classification of Diseases, Tenth Revision* [*ICD-10*] classification M20.1) and an operative code for first metatarsal osteotomy (NHK30 by NOMESCO classification of surgical procedures) during 2019-2022, and the analysis for this study was conducted in 2023. The median time from the day of the operation to the last review of the charts was 70.4 (range 18-107.5) months. During 2013-2018, there were 740 procedures for 659 patients. Thirty operations were revisions for residual hallux valgus deformity, and 25 procedures had an incorrect operative code. The total number of excluded procedures was 55, leaving 685 procedures for analysis (Figure 1). Median age was 55 (range, 13-87) years, 597 (87%) of the patients were female, and median BMI was 26 (n = 648; range, 15-47). First, metatarsal osteotomies were divided into distal (chevron and Mitchell), midshaft (scarf), and proximal (medial open wedge osteotomy) osteotomies. The operating surgeon had determined the choice of osteotomy type for each case. Most of the distal osteotomies were fixed with bioabsorbable pins or cannulated screws, the midshaft with cannulated screws, and the proximal with proximal plates. Surgeon’s experience was classified into 3 groups by consensus as follows: the first group included orthopaedic residents with the orthopaedic surgeon as an assistant (n = 90, 13%), the second group general orthopaedic surgeons (n = 383, 56%), and the third group orthopaedic surgeons with years of experience in and practicing only foot and ankle surgery (n = 212, 31%). None of the authors were among the groups of orthopaedic surgeons. Most surgeries, 520 (75.9%), were performed in the University hospital, and 165 (24.1%) in district hospitals of the region. Demographic data are shown in Table 1.

**Table 1.** Demographic Data of Patients.^a^

	All Operations	Reoperations	Not Reoperated	*P*
All	685	79 (11.5)	606 (88.5)	
Female	597 (87)	64 (81)	533 (88)	.0829
Male	88 (13)	15 (19)	73 (12)	
Age, y, median (range)	55 (13-87)	54 (13-74)	56 (16-87)	.3116
BMI, median (range)	26 (15-47)	26 (25-38)	26 (15-47)	.8845
Diabetes mellitus	34 (3)	9 (11)	25 (4)	.0052
Smoking	70 (10)	9 (11)	9 (1.5)	.7179
Rheumatoid arthritis	18 (3)	4 (5)	14 (2)	.1502
Type of osteotomy				<.0001
Distal	387 (56)	19 (24)	368 (76)	
Shaft	257 (38)	48 (61)	209 (39)	
Proximal	41 (6)	12 (15)	29 (85)	
Preoperative HVA, degrees, median (range) (n = 669)	28 (0–52)	32 (12–50)	28 (0-52)	<.0001
Postoperative HVA, degrees, median (range) (n = 444)	14 (-8–48)	16.5 (5-48)	14 (-8–44)	.0137
Preoperative IMA, degrees, median (range) (n = 669)	15 (5–30)	16 (7–24)	15 (5-30)	.0551
Postoperative IMA, degrees, median (range) (n = 443)	8 (0–24)	8 (3–20)	9 (0-24)	.9707
Experience of operating physician,				.6319
Resident	90 (13)	8 (10)	82 (14)	
General orthopaedic surgeon	383 (56)	44 (56)	339 (56)	
Foot and ankle surgeon	212 (31)	27 (34)	185 (30)	
Hospital				.0189
Central (1 hospital)	520 (76)	59 (75)	461 (76)	
District (4 hospitals)	165 (24)	20 (25)	145 (24)	

Abbreviation: HVA, hallux valgus angle; IMA, intermetatarsal angle

a Unless otherwise noted, values are n (%). *P* values for all variables were tested for association with reoperation in univariate analysis.

### Radiologic analysis

Hallux valgus angle and intermetatarsal (IMA) angles were measured pre- and postoperatively from weightbearing dorsoplantar radiographs of the foot by 2 orthopaedic surgeons with at least 2 years of experience in orthopaedic surgery. Nonweightbearing radiographs were excluded from the alignment analysis. HVA was measured by 2 longitudinal bisections of the shafts of the first metatarsal and the proximal phalanx of the hallux. The median preoperative HVA was available from 669 patients and was 29 (range, 0-52) degrees. Seven cases with a normal preoperative HVA had mild to moderate preoperative IMA. The median postoperative HVA was available from 444 patients and was 15 (range, –8 to 48) degrees. IMA was measured by 2 longitudinal bisections of the first and second metatarsal shafts. The median preoperative IMA was available from 669 patients and was 15 (range, 5-30) degrees, and the median postoperative IMA was available from 443 patients and was 9 (range, 0-24) degrees.

### Statistical Analysis

Because of asymmetrical distribution, the median, interquartile range (Q1, Q3), and range were reported for continuous variables. Categorical variables were summarized with counts and percentages. Associations between reoperation (2-class categorical variable, yes or no) and variables (sex, age, BMI, smoking, surgeon’s experience, diabetes, rheumatoid arthritis, preoperative HVA, preoperative IMA, postoperative HVA, postoperative IMA, hospital, type of osteotomy, follow-up time) were studied one by one with Kruskal-Wallis test and χ^2^ test (for categorical variables). The Kruskal-Wallis test was used to study the associations between HVA or IMA and the surgeon’s experience and type of osteotomy. The association between reoperation and explanatory variables was analyzed using logistic regression. The model included sex, age, BMI, smoking, surgeon’s experience, hospital size, type of osteotomy, preoperative HVA, diabetes, and rheumatoid arthritis. Sensitivity analysis was performed by changing the preoperative HVA to postoperative HVA in the model. Odds ratios (ORs) with 95% Wald CIs were calculated.

The normality of variables was evaluated visually and tested with the Shapiro-Wilk test. Because of the non-normality of the continuous variables, nonparametric methods were used. All tests were performed as 2-sided with a significance level set at .05. The analyses were conducted using the SAS system, version 9.4, for Windows (SAS Institute Inc, Cary, NC).

## Results

**Figure 2.**
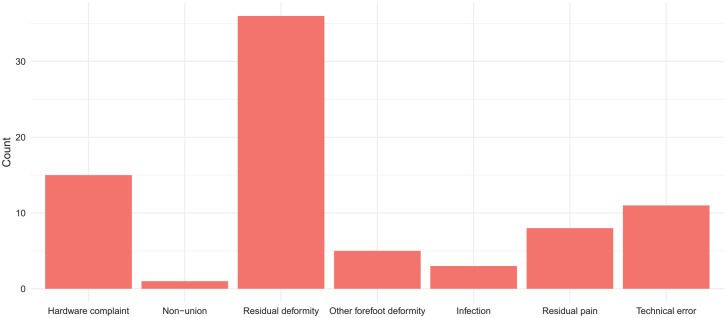
The numbers and reasons for reoperations.

There were 79 first reoperations (11.5%) at a median of 14 months (range 1-83) postoperatively. Six patients had multiple reoperations (Table 2). The reasons for reoperations were divided into 7 categories: hardware removal, nonunion, residual deformity, other forefoot deformity, infection, residual pain, and technical error. A residual deformity was considered when postoperative HVA was above normal (<15°), and the indication for the reoperation was defined as a residual or recurrent deformity by the operating surgeon. A technical error was considered in cases with a clear technical error, but other categories were not applicable, for example, the hardware inside the joint or osteonecrosis related to an incorrect osteotomy line. Fifteen reoperations were merely hardware removals. Most reoperations (46%) were performed because of residual deformity. The numbers and reasons for reoperations are presented in Figure 2. The different reoperations are shown in Table 3, the reasons for reoperations by the experience level of the surgeon in Table 4, the reasons for reoperations divided by pre- and postoperative HVA and IMA in Table 5, and the pre- and postoperative HVA and IMA divided by osteotomy types and by surgeons’ experience level in Tables 6 and 7, respectively.

**Table 2.** Patients With Multiple Revision Operations.^a^

Patient	Comorbidities	Primary Operation	First Revision			Second Revision			Third Revision			Fourth Revision		
			Time	Reason	Type	Time	Reason	Type	Time	Reason	Type	Time	Reason	Type
1	Sarcoidosis, asthma	Distal osteotomy (chevron)	12	Nonunion	Revision with autologous bone graft	24	Nonunion	I MTPJ arthrodesis						
2		Midshaft osteotomy (scarf)	30	Residual pain	II MT Weil	44	Residual deformity	I TMTJ arthrodesis						
3		Midshaft osteotomy (scarf)	15	Hardware complaint	Implant removal	80	Residual pain	Second implant removal						
4	Rheumatoid arthritis	Distal osteotomy (chevron)	10	Residual pain	Implant removal	16	Osteonecrosis	I MTPJ arthrodesis						
5	Rheumatoid arthritis, diabetes mellitus (type 1)	Midshaft osteotomy (scarf)	9	Residual deformity	Distal osteotomy (Mitchell)	21	Nonunion	Revision	59	Other forefoot deformity	MT II-IV Weil osteotomies			
6		Midshaft osteotomy (scarf)	5	Technical error	FHL re-insertion	24	Technical error	I MTPJ arthrodesis	31	Nonunion	Revision I MTPJ arthrodesis	34	Infection	Implant removal, revision, antibiotics, multiple revisions, painful pseudoarthrosis

Abbreviations: FHL, flexor hallucis longus; MT, metatarsal; MTPJ, metatarsophalangeal joint; TMTJ, tarsometatarsal joint.

a Time is recorded as months from index operation.

**Table 3.** Type of Revision Surgery According to the Indication of Revision Operation.^a^

	Type of Revision Surgery, n							
Reason for Revision Surgery	Implant Removal	Arthrodesis First MTPJ	Reosteotomy	Arthrodesis First TMTJ	Wound Revision	Forefoot Amputation	Other	Total
Hardware complaint	14	0	0	0	0	0	1	15
Nonunion	0	0	1	0	0	0	0	1
Residual deformity	0	19	12	4	0	0	1	36
Other forefoot deformity	0	0	0	0	0	0	5	5
Infection	2	0	0	0	1	0	0	3
Residual pain	2	2	0	0	0	0	4	8
Technical error	8	0	0	0	0	1	2	11
Total	26	21	13	4	1	1	13	79

Abbreviations: MTPJ, metatarsophalangeal joint; TMTJ, tarsometatarsal joint.

a If there were several operations on one foot, it appears only in the column of the last operation, but the same case may appear on several rows.

**Table 4.** Reasons for Reoperations by Experience Level of the Surgeon.^a^

	Reason for Reoperation							
Surgeon	Hardware Complaint	Nonunion	Residual Deformity	Other Forefoot Deformity	Infection	Residual Pain	Technical Error	Total
Foot and ankle surgeon	11 (41)	0	7 (26)	1 (4)	1 (4)	2 (7)	5 (18)	27
General orthopaedic surgeon	3 (7)	1 (2)	26 (59)	4 (9)	2 (5)	4 (9)	4 (9)	44
Resident	1 (12)	0	3 (38)	0	0	2 (25)	2 (25)	8
Total, n	15	1	36	5	3	8	11	79

a Data are expressed as total numbers and percentage (in parentheses) of all reoperations of the group.

**Table 5.** Reasons for Reoperations by the Osteotomy Type.^a^

	Reason for reoperation							
Osteotomy Type	Hardware Complaint	Nonunion	Residual Deformity	Other Forefoot Deformity	Infection	Residual Pain	Technical Error	Total
Distal osteotomy	2 (10)	1 (5)	7 (37)	3 (16)	0	3 (16)	3 (16)	19
Shaft osteotomy	11 (23)	0	21 (44)	1 (2)	2 (4)	5 (10)	8 (17)	48
Proximal osteotomy	2 (17)	0	8 (67)	1 (8)	1 (8)	0	0	12
Total	15	1	36	5	3	8	11	79

a Data are expressed as total numbers and percentage (in parentheses) of all reoperations of the group.

**Table 6.** Median Preoperative and Postoperative HVA and IMA (Degrees) by Osteotomy Type.

	Distal	Shaft	Proximal	*P*
Preoperative HVA, median (range), n = 669	26 (10-48)	31 (15-50)	34 (0-52)	<.0001
Postoperative HVA, median (range), n = 444	14 (1-44)	13 (-8-48)	18 (2-42)	.1282
Preoperative IMA, median (range), n = 669	14 (5-24)	16 (8-30)	18 (9-23)	<.0001
Postoperative IMA, median (range), n = 443	9 (2–20)	8 (1-24)	9 (0-14)	.7005

Abbreviations: HVA, hallux valgus angle; IMA, intermetatarsal angle.

a The *P* values were calculated using the Kruskal-Wallis test.

**Table 7.** Median Preoperative and Postoperative HVA and IMA (Degrees) by the Experience Level of the Surgeon.

	Resident	General Orthopaedic Surgeon	Foot and Ankle Surgeon	*P*
Preoperative HVA, median (range), n = 669	26 (16-50)	30 (0-52)	26 (10-51)	<.0001
Postoperative HVA, median (range), n = 444	15 (4-37)	16 (-8-48)	11 (-3-35)	<.0001
Preoperative IMA, median (range), n = 669	15 (8-25)	16 (7-30)	15 (5-26)	.0191
Postoperative IMA, median (range), n = 443	10 (4–24)	9 (2-21)	8 (0-20)	.0038

Abbreviations: HVA, hallux valgus angle; IMA, intermetatarsal angle.

a *P* values were calculated using the Kruskal-Wallis test.

### Factors Associated With Short-term Revision Operations in Univariate Analysis

In univariate analysis, a higher preoperative HVA (*P* < .0001), a higher postoperative HVA (*P* = .0137), diabetes (*P* = .0052), and type of osteotomy (*P* < .0001) were statistically significant risk factors for all-cause reoperation, whereas age, sex, BMI, smoking, rheumatoid arthritis, preoperative IMA, postoperative IMA, surgeon’s experience, hospital size, and follow-up time were not (all *P* > .05). Overall, 4.9% of distal, 18.7% of midshaft, and 29.3% of proximal osteotomies were reoperated, respectively.

### Factors Associated With Revision Operations in Multivariate Analysis

In multivariate analysis, a higher preoperative HVA (*P* < .0110), diabetes (*P* = .0023), and type of osteotomy (*P* < .0001) were similarly associated with all-cause reoperation as in the univariate analysis. The results remained unchanged when preoperative HVA was changed to postoperative HVA. The odds ratio for reoperation for proximal osteotomy was 8.7 (95% CI 3.4-22.2) and for midshaft osteotomy 4.4 (95% CI 2.3-8.4) compared with distal osteotomy, respectively.

## Discussion

We found a relatively high number of short-term reoperations after hallux valgus deformity surgery with first metatarsal osteotomies. Higher preoperative HVA, diabetes, and type of osteotomy were statistically significantly associated with all-cause reoperation both in univariate and multivariate analysis. However, these associations may be partly driven by confounding by indication because more severe deformities tended to receive proximal or midshaft osteotomies. Higher postoperative HVA was also statistically significantly associated with all-cause reoperation in univariate analysis; however, as half of the reoperations were performed because of residual deformity, as defined by postoperative HVA, the association is not necessarily true. The results of the multivariate analysis remained unchanged when preoperative HVA was changed to postoperative HVA, supporting the robustness of the association despite missing radiographs.

In the most recent studies, the incidence of revision surgery has been lower, and the time between the index operation and the revision procedure is longer than in our study. In a study by Wagner et al^18^ with 144 patients (187 feet) with mean preoperative HVA of 35.6 degrees operated with proximal first metatarsal osteotomy in a median follow-up time of 35 months (range, 12-73), there were 12 feet (6.4%) with severe recurrence of the deformity requiring revision surgeries. Removal of hardware was needed in 23 feet (12.3%) for symptomatic hardware and 5 feet (2.6%) developed hallux varus, of which 2 required surgery.^18^ In a large study cohort with chevron osteotomy for hallux valgus correction with mean preoperative HVA of 28.5 degrees, 12.6% of the patients had a reoperation for some cause.^15^ In a study addressing the radiologic outcome of 32 scarf and 181 chevron osteotomies with preoperative HVA of 39.55 and 31.3 degrees, respectively, in a follow-up period of over 3 years, the reoperation rates were 3.3% in chevron osteotomy patients and 6.3% in scarf osteotomy patients with no significant differences between groups.^19^ In a multicenter retrospective review of 646 patients operated for hallux valgus deformity, the total rate for reoperation was 5.56% for chevron-Austin osteotomy, 8.82% for closing base wedge osteotomy, and 8.19% for modified Lapidus arthrodesis in a follow-up time of 1-4 years. The reoperation rates were 1.85%, 2.92%, and 2.94% for recurrent hallux valgus, respectively.^17^ In the review by Barg et al,^8^ the mean rate of secondary surgery other than hardware removal was 2.1% (range, 1.4%-3.0%), and the mean rate of hardware removal was 3.8% (range, 2.2%-5.9%). The mean overall follow-up time of the studies regarding osteotomies varied from 1.9 to 6.0 years.^8^ In an extensive retrospective analysis with a total of 22 199 feet, including first metatarsal osteotomies, first MTP or TMT joint arthrodesis, or other procedures, there was an all-cause revision rate of 5.6% in the osteotomy group and 6.4% in the arthrodesis group (MTP and TMT).^11^ Multiple comorbidities were correlated with higher revision rates. During the study period, the odds of hallux valgus revision rates were significantly greater in procedures performed before 2010.^11^ In a recent review by Migliorini et al^12^ addressing the results of revision after failed hallux valgus surgery with 586 procedures in 569 patients, the time between index and revision surgery was 95.7 ± 36.6 months. The revision after failed hallux valgus surgery yielded satisfactory results, although a further 5.1% recurrences and 4.1% nonunions were detected.^12^ In addition, further surgical procedures were detected in 8.7% of the cases, of which two-thirds were hardware removals.^12^

The amount of technical error and residual deformity was high in this series. There are multiple causes for the failure of primary hallux valgus deformity correction, including patient-related and surgical factors, the most important of which is probably inadequate patient and procedure selection. Choosing the appropriate procedure to address the given hallux valgus pattern, the technical competency of performing the corrective procedure, and recognizing the need for additional procedures to prevent failures.^20^ In this series, the type of osteotomy was strongly related to the risk of reoperation as only 4.9% of the distal osteotomies were reoperated compared with 18.7% of the midshaft and 29.3% of the proximal osteotomies, respectively. The higher technical demand for more proximal osteotomies than distal ones likely explains this. Additionally, it can be discussed whether, in some cases, with a proximal osteotomy, a modified Lapidus procedure could have been a more suitable option. However, in a meta-analysis including 10 RCT studies and 793 feet with 6-96 months’ follow-up comparing the results of different types of osteotomies, there were no differences between distal and midshaft osteotomies regarding complications and recurrence, and the results were unclear between distal and proximal osteotomies.^21^ Although it was previously shown that foot and ankle surgeons could achieve better radiologic results in hallux valgus correction by first metatarsal osteotomy compared with general surgeons and residents,^22^ the experience level of the operating surgeon was not statistically significantly associated with the all-cause reoperation rate. However, most of the reoperations after deformity correction by foot and ankle surgeons were merely hardware removals compared with general orthopaedic surgeons, who had more recurrence of the deformity (Table 4). The preoperative severity of deformity was lower in patients operated on by the foot and ankle surgeons, but they reached a significantly better postoperative result compared with other surgeons (Table 7).

The severity of different indications for reoperation varies. An isolated hardware removal can be considered as a mild indication for reoperation and was encountered when there was, for example, a palpable screw head or the patient just wanted the metal removed. The cases with a misplaced screw, for instance, inside the joint, were classified as a technical error category, and they can be interpreted as a more severe reason. Nonunions and infections can result from surgical- or patient-related issues, and the percentage of those in this series was low, like in previous reports. In a review study by Barg et al,^8^ the mean incidence of infections in the included studies was 2.6% (range, 1.5%-11.4%) and of nonunion 0.04% (range, 0%-3.8%) compared with our results of 3.8% and 1%, respectively.

Diabetes is generally considered a risk factor for wound healing complications, infection, and nonunion. Still, interestingly, in this series, it appeared to be associated with all indications for revision surgery. Most cases were operated on because of residual deformity, and only 1 was for infection. A definitive analysis of the reasons was not possible because of the small number of cases overall. Additionally, because of the retrospective nature of this study, one must exercise caution when extrapolating these results, as the data regarding diabetes may be subject to bias.

Previously, smoking has been associated with an increased risk of nonunion in Lapidus procedures and a higher risk of wound complications in all hallux valgus deformity correction procedures.^12,23^ In this series, smoking was not associated with a higher risk of revision surgery. This might be explained by the fact that not all wound complications or nonunions require a revision operation. In addition, the smoking status is only sometimes reliably obtained from patient data, which might result in bias in the analysis. Rheumatoid arthritis was not identified as a risk factor for revision surgery, which at least 2 things might explain. First, another procedure than a first metatarsal osteotomy, like a first MTPJ arthrodesis, might have been primarily chosen for rheumatoid patients, and second, some of the rheumatoid patients might be recorded only by diagnostic code of rheumatoid arthritis and not by hallux valgus, which was not taken into consideration in the data search.

### Limitations of This Study

One limitation of this study is the retrospective setting. The design introduces selection bias and confounding by indication (eg, surgeons preferentially chose proximal osteotomies for more severe deformities), which we could not fully adjust for. In addition, although this was quite a large series, only some of the patients requiring revision surgery were necessarily available for the analysis, as some of them might have continued their treatment elsewhere. Approximately one-third of postoperative radiographs were missing, and no interobserver reliability statistics were recorded, which may have biased the radiographic analyses. The radiologic analysis also did not include an assessment of sesamoid-metatarsal reduction. The study's strength is that it represents true life involving different hospitals, mixed patient material, and surgeons of every level.

## Conclusion

In this large series, the incidence of short-term reoperations (median 14 months) after surgical correction of hallux valgus deformity with first metatarsal osteotomy was higher than in most previous studies. The preoperative and postoperative HVA, diabetes, and type of osteotomy were associated with revision surgery regardless of the reason for reoperation. Given the observational design, potential confounding, and incomplete follow-up, these associations should be interpreted cautiously, We could not identify a patient-level explanation for the higher reoperation rate relative to previous reports. Regarding the high number of recurrences and technical errors as an indication for reoperation, the reason is probably more associated with surgical rather than patient-related factors. This is also supported by the finding that technically more demanding osteotomies had a significantly higher risk for short-term reoperations.

## Supplemental Material

sj-pdf-1-fao-10.1177_24730114251359646 – Supplemental material for Incidence and Risk Factors for Short-Term Reoperations After Open First-Metatarsal Osteotomy for Hallux Valgus Supplemental material, sj-pdf-1-fao-10.1177_24730114251359646 for Incidence and Risk Factors for Short-Term Reoperations After Open First-Metatarsal Osteotomy for Hallux Valgus by Tuuli Erjanti, Heli Keskinen, Tiia Rissanen, Keijo Mäkelä, Petteri Lankinen, Inari Laaksonen and Helka Koivu in Foot & Ankle Orthopaedics
